# Inequalities in Home Learning and Schools’ Remote Teaching Provision during the COVID-19 School Closure in the UK

**DOI:** 10.1177/00380385221122444

**Published:** 2022-10-28

**Authors:** Sait Bayrakdar, Ayse Guveli

**Affiliations:** King’s College London, UK; University of Essex, UK

**Keywords:** COVID-19, distance teaching, education, ethnicity, home schooling, parental background, remote teaching

## Abstract

Millions were affected by COVID-19 school closures, with parents and schools caught unprepared. Education is expected to play a role in creating equal opportunities, so transferring schooling responsibilities to families may have increased learning inequalities generated by family backgrounds. We examined the time students spent on home learning and explored the role of the schools’ distance teaching provision in explaining differences traditionally attributed to parental education, eligibility for free school meals, ethnic background and single parenthood. Using the Understanding Society COVID-19 dataset, we found children who received free school meals, single-parent families and children with parents with lower formal education qualifications and Pakistani or Bangladeshi backgrounds spent significantly less time on schoolwork. However, schools’ provision of offline and online distance teaching and homework checking significantly increased the time spent on learning and reduced some inequalities, demonstrating the policy relevance of digital preparedness to limit learning loss in school closures.

## Introduction

The COVID-19 pandemic transformed homes into classrooms in a matter of weeks around the world. The learning environment radically changed, and many schools had to create distance teaching resources overnight. UK schools closed on 20 April 2020 for all students except children of key workers and children with special needs. While many schools tried to deliver learning materials in the form of online teaching sessions, online and offline tasks and homework, provision was not universal. Inequalities quickly attracted attention, with special concerns about marginalised students with limited resources available to them (Engzell et al., 2020; UNESCO, 2020; Van de Werfhorst et al., 2020).

Home learning may have generated new inequalities and exacerbated existing ones; alternatively, schools’ involvement in home learning and provision of distance teaching may have mitigated disparities. We investigated whether parental background was linked to inequalities in the time students spent on schoolwork during the 2020 lockdown; rather than simply accepting the general understanding of a link between parental background and learning inequalities, we also considered the effect of schools’ provision of learning opportunities on these inequalities. Since we had no direct measure of learning during the lockdown, we used the amount of time students spent on home learning each day as an indicator of learning.

Education is an important determinant of an individual’s life prospects (Hout and DiPrete, 2006; Machin, 2006). Schools develop talents and abilities and play a key role in equalising opportunities by providing arguably the most direct route for social advancement. That said, research shows formal education does not completely level the playing field (Breen, 2004) and schools more often reward children from families with relatively higher socio-economic and cultural capital and marginalise others (Archer, 2008; Bourdieu, 2002; Collins, 2009). When schools are closed and unable to deliver learning (or deliver it in an unsystematic and selective manner), and teaching responsibilities are transferred to families, existing social inequalities in learning may be amplified because the extent to which children can continue learning at home may depend on the resources available to the family.

Recent literature shows examples of losses in learning during the pandemic-related school closures for all children (Burgess and Sievertsen, 2020), with more losses among working-class families (Andrew et al., 2020; Bayrakdar and Guveli, 2020a; Engzell et al., 2020). Drawing on these studies, we examined the time spent on learning by parental socio-economic characteristics, ethnicity and single parenthood. Using information from parents on schools’ provision of distance teaching, we also investigated whether schools’ involvement might explain disparities in home learning. Our findings showed that schools’ remote teaching provision explained a considerable part of the inequalities in home learning, thus pointing to the failures of the institutions and school systems as the main source of inequalities.

## Family Background and Educational Inequalities

Scholars have often scrutinised the role of schools in societies. Some consider schools are the engines of social and cultural reproduction (Bourdieu, 2002; Collins, 2009), while others contend schools fuel equal opportunities in societies. On the one hand, formal education provides young people with the information and skills needed to participate in society and succeed in the labour market. Schools are seen as the main mechanism of social mobility, allowing marginalised individuals to improve their social and economic status (Machin, 2006). The classic social mobility literature suggests the impact of parental background on educational attainment and other life prospects will decrease, and talent, skills and educational credentials will become more decisive over the course of industrialisation (Blau and Duncan, 1967; Coleman et al., 1966; Erikson and Goldthorpe, 2010; Guveli, 2006; Machin, 2006). Accordingly, social policies aim to minimise the impact of family background on education, to ensure equal opportunities for all members of society regardless of social or family background (Morgan et al., 2006; Roemer, 1998). Schools seek to prepare, select and distribute children into privileged middle-class positions, thus supposedly functioning as a meritocratic social force.

On the other hand, education systems and policies are recognised as sources of inequality, maintaining and reinforcing the interests and privileges of the middle classes (Bourdieu, 2002; Collins, 2009; Gillborn, 2005; Ladson-Billings, 1998). Studies find prejudice and discriminatory processes engrained in the education system and school culture adversely affect students with working-class, ethnic minority and atypical family backgrounds (Archer, 2008; Bokhove and Hampden-Thompson, 2021; Van den Bergh et al., 2010). The so-called meritocratic systems are likely to favour those who have the privilege to define the merits; as such, they may preserve social inequalities and support the decisive role of parental resources in formal educational attainment over time (Bayrakdar and Guveli, 2020b; Breen, 2004; Mijs, 2016; Rotman et al., 2016).

The UK is no exception. At various key stages of education, students from working-class families acquire disproportionately lower qualifications, follow less privileged career routes and enter less prestigious universities than their more privileged peers (Bukodi and Goldthorpe, 2013; Heath and Clifford, 1990; Sullivan et al., 2013). Recent administrative data indicate children entitled to free school meals (FSMs) because of family poverty have 18–20% lower attainment than their more advantaged peers (Social Mobility Commission, 2019).

There are also differences for some (but not all) ethnic minority groups. For example, students with Black-African, Black-Caribbean, Pakistani and Bangladeshi backgrounds, on average, have lower grades than their White comparators, even though these gaps have diminished in the last decade (DfE [Department for Education], 2015; Rothon, 2007). Once socio-economic resources are controlled, the formal education gap for Black-African, Black-Caribbean, Bangladeshi and Pakistani students is reduced, suggesting ethnic inequalities are intertwined with social class inequalities in creating unfavourable outcomes for these students. Some scholars explain the remaining gap by pointing to discrimination, racism and other forms of unfair treatment, as well as schools’ failure to engage with diverse communities (Baysu et al., 2021; Heath and Birnbaum, 2007). Such inequalities suggest schools and educational policies, to a large extent, fail to provide inclusive practices and marginalise and racialise ethnic minority students.

These systemic problems were likely to have worsened during the lockdown, with a patchy and stripped-down version of teaching provision better serving some than others. Those with socio-economic privileges were more likely to acquire additional support as needed, to have adequate space, Internet and technological equipment, possibly exacerbating existing learning inequalities.

### Learning and School Provision

Most formal learning takes place in schools. When schools are not open, learning is disrupted. Many studies suggest any interruption in schooling results in a loss of learning (Alexander et al., 2001; Burgess and Sievertsen, 2020). A meta-analysis of learning loss during summer vacation suggested that, on average, one month of learning is lost (Cooper et al., 1996). Burgess and Sievertsen (2020) studied the impact of short-term school closure due to previous pandemics, natural disasters and school strikes and found 12 weeks of schooling interruption drops test scores significantly.

The effect of learning loss is greater for some groups than others. Alexander et al. (2001) suggested children from underprivileged backgrounds experience learning losses during school closures while their well-off comparators continue to explore and learn. Cooper et al. (1996) found the test scores of students from working-class families decreased after summer vacation, but the test scores of those from middle-class families increased.

By the same token, the inability of schools to provide adequate remote teaching during the pandemic might have increased social inequalities and widened the education gap, as more privileged families could compensate for the disruption through financial and other kinds of resources. The hardest-hit children were likely to be those from low-income families, those in single-parent families and those with ethnic minority/migration backgrounds, as their needs and household circumstances may not have been addressed or even acknowledged by the education system during the pandemic.

### Learning Loss during the COVID-19 Pandemic

A learning loss was expected during the pandemic because of the school closures, possibly setting back students’ cognitive gains in the long term (Bol, 2020) and disproportionately affecting underprivileged children (Azevedo et al., 2020; UNESCO, 2020). Emerging research suggests far-reaching educational consequences, especially for children in working-class families (Andrew et al., 2020; Bol, 2020; Engzell et al., 2020). For example, researchers found a significant learning loss during the eight-week school closure in the Netherlands, a country with one of the best infrastructures for remote teaching (Engzell et al., 2020). Learning loss might be considerably greater in countries with a longer period of school closure and less preparedness for remote teaching provision, such as the UK (Van de Werfhorst et al., 2020).

Pre-pandemic studies of formal educational attainment and inequalities in the UK reveal substantial penalties for working-class families and certain ethnic groups (Pensiero and Schoon, 2019; Stevens et al., 2019). For example, children from low-income families may live in small houses with no suitable place to do their schoolwork without distraction. They might not have Internet or IT facilities; if they do, they will likely need to share them with parents or siblings. School interruption is likely to widen the gap if these children whose needs are already known are underserved. While some studies (Andrew et al., 2020; Pensiero et al., 2020) have already documented a pandemic-related learning loss for students from working-class backgrounds, the researchers did not examine the impact of school closure on the learning of children with ethnic minority backgrounds and the poorest students (e.g. those receiving FSMs), and these students also seem likely to experience marginalisation and exclusion during school closures. In contrast, families from privileged backgrounds might compensate for the negative impact of school closure with their resources and knowledge, including their ability to negotiate with service providers to maintain their advantage (Andrew et al., 2020; Bol, 2020). Students from middle-class families may even make greater strides, as their parents might be able to spend more time with their children, teach them individually and/or outsource support to online tutors to help with schoolwork (Boonk et al., 2018; Calarco, 2018; Hastings and Matthews, 2011; Lareau, 2011).

Beyond socio-economic status, parental education is another possible factor in the effects of school closure: parents with lower levels of formal qualifications may feel less able to take up teaching responsibilities. Looking at the Netherlands, Bol (2020) found parents with lower levels of formal educational qualifications reported feeling less confident supporting their children’s learning during the 2020 lockdown or said they had limited understanding of the material schools provided during the closure (Bol, 2020; Cullinane and Montacute, 2020).

Parents’ working patterns can have an impact on their children’s home learning as well. Those working long hours during the pandemic, particularly outside the home, might have had less time and energy to support their children’s studies and well-being. School closures might have placed additional burdens on single parents as they might have more competing responsibilities in and outside the home. Using the Understanding Society COVID-19 data, Benzeval et al. (2020) found single parents faced more economic loss than others during the lockdown. Single parents might also have been less able to dedicate time and resources to home school their children.

The UK’s ethnic minorities suffered relatively more than others during the pandemic (Benzeval et al., 2020), and this may have extended to education, with school closures widening the existing gaps between minority and majority students. Recent research shows those with Pakistani, Bangladeshi, Black-African and Black-Caribbean backgrounds are more likely to be key workers, thus rendering them vulnerable to the virus and putting extra pressure on family life (Platt and Warwick, 2020). Ethnic minority parents might be less able to support their children’s learning during school closures for other reasons as well. While their aspirations for success may be high, some may have limited knowledge of the education system, curricula and teaching style (Bayrakdar and Guveli, 2020b). Fewer opportunities to communicate with schools may create an unfavourable environment for learning, especially if schools do not actively create inclusive practices and reach out to all parents (Crozier and Davies, 2007). If families are not served by the education system at the best of times, they might need to rely on their social capital to support their children’s learning during more difficult times, and this may be limited (Bayrakdar, 2015).

### Schools’ Home-Schooling Provision

Schools have the responsibility to foster talent, skills and cognitive competences and to provide knowledge transfer in our knowledge society. They are expected to enhance the chances of status attainment for all children and are considered the engine of social mobility, safeguarding equal opportunities in the labour market and beyond. Schools’ involvement during the pandemic lockdown had the potential to provide learning continuity, increase students’ motivation to do schoolwork and mitigate learning drawbacks of the shutdown. Even if physically closed, schools could offer online classes, materials, supervision and other kinds of communication to minimise the disruptive effect of closure. When students are given the right materials, their learning may be less disrupted. Thus, the provision of learning materials could maintain schools’ equalising function and possibly even reduce learning gaps.

However, schools might differ in their provision of distance teaching and home learning guidance, depending on staffing, IT facilities and distance teaching knowhow. We did not expect remote teaching would fully mitigate the negative effects of school shutdown and associated learning loss, and we further argued schools’ home-schooling provisions might even explain part of the inequalities in learning, which are more generally attributed to family background.

Even in normal times, schools’ resources, funding and teacher quality differ, and these characteristics affect student outcomes (Levačić and Vignoles, 2002; Rivkin et al., 2005; Steele et al., 2007). Education policies have long been criticised for ignoring the needs of students from minority backgrounds and more often serving the middle-class White majority (Gillborn, 2005). In addition, the education system in the UK has been transformed by neo-liberal ideals, with schools forced to compete in an ‘education market’ wherein accountability and responsibility have shifted from the education system to individual schools (Ball et al., 1996; Hursh, 2005). For example, school performance tables are published annually, and parents often base their schooling decisions on them (Perryman et al., 2011). The creation of a ‘quasi-market’ in education affects student intake, as parents with more resources send their children to better schools (Hobbs, 2016; West and Pennell, 2010), while others may have unqualified, inexperienced and out-of-subject teachers, possibly in schools with poor staff retention (Allen and Sims, 2018). Research shows that in England, teachers at schools with a high proportion of underprivileged students attend ICT-related professionalisation activities less often than their counterparts in schools with well-off students (Van de Werfhorst et al., 2020).

## Study Hypotheses

Considering these factors, we expected some home learning differences between select populations of students (working-class families, single-parent families, ethnic minorities) could be explained by variations in schools’ remote teaching support during the 2020 lockdown. We also expected home learning disparities would be mitigated if all students received the same distance teaching provision. We formulated the following three research questions and hypotheses:

1. How much time did students spend on schoolwork during the COVID-19 school closure in the UK?
2. To what extent did the amount of time students spent on schoolwork during the school closure differ by parental socio-economic characteristics, ethnicity and single parenthood?
3. To what extent did schools’ provision of remote teaching (online and/or offline home learning material; checking homework) explain inequalities in the amount of time spent on homework across students with different family backgrounds?

We expected students, on average, would spend less time on schoolwork during the school closure than during regular learning periods (Hypothesis 1). We also expected students from working-class, ethnic minority and single-parent households and those whose parents had lower formal educational qualifications would spend less time on schoolwork than their comparators in other student groups (Hypothesis 2). Finally, we expected differences in schools’ provision of distance teaching would explain some of the observed differences in the amount of time spent on homework across social class, ethnic background and family composition (Hypothesis 3).

## Method

### Data

We used the first wave of the Understanding Society COVID-19 Study (University of Essex, 2020). Understanding Society is the UK’s main longitudinal household survey, with information on all adults living in 40,000 households. The Understanding Society COVID-19 Study is a panel study documenting the experiences of the UK population during the pandemic. The first wave was fielded in April 2020 during the first lockdown when schools were closed for most students. The sample for the COVID-19 Study included all active members of the main Understanding Society Study, as well as immigrant and ethnic minority boost samples. In this sense, it differs from many COVID-19 surveys, as it used a probability sample generated by a major household panel study. The representativeness of the data has been evaluated frequently; the high-quality data allow population inferences (Benzeval et al., 2020; Fisher et al., 2019).

Participants were invited to answer questions taking approximately 20 minutes on the web and were offered a small financial incentive. A pre-notification letter was sent to their postal address on 17 April 2020, followed by three reminders to their email address and/or phone number during the fieldwork period. Overall, 46.7% of those participating in the ninth wave of Understanding Society (the last wave before the pandemic) participated in the COVID-19 Study. Survey response rates fell substantially during the pandemic, and the response rate of the panel survey was lower than previous waves, but it was still better than many other COVID-19 surveys (Dahlhamer et al., 2021).

If respondents had a child or children living in the household for whom they were the parent or guardian, they were asked the questions in the home-schooling module in the first wave of the Understanding Society COVID-19 web survey; these data cover children attending primary, secondary and higher secondary school. We used the children’s dataset; units of analysis were the children. We matched parental characteristics from the main Understanding Society COVID-19 wave 1 and the baseline Understanding Society wave 9 datasets.

After removing cases with missing information, 3867 children were left. Wave 9 of the Understanding Society dataset does not include all adults who participated in the COVID-19 survey, so inclusion of parental information from the main Understanding Society data reduced the number of cases to 3150. As a robustness check, we ran the same models but excluded the information from the main Understanding Society dataset; this did not change our conclusions (online Appendix A).

### Selectivity

We focused on students who were not in school at the time of the survey and who received schoolwork from their schools; this might mean a selective group of students in terms of socio-economic and ethnic background. We noted the distribution of these variables because families eligible for FSMs and those of certain ethnic minority heritage are likely to be overrepresented in key workers’ jobs (Platt and Warwick, 2020); thus, their children might have continued to attend school more often during the school closure.

In Tables 1 and 2, we show children who did *not* attend school and did *not* receive schooling material or online teaching from school for home learning, and children who were *not* at school and received schooling material or online teaching from school by eligibility for FSMs (Table 1) and parental ethnic background (Table 2).

**Table 1.** Free School Meal (FSM) by still attending school; not attending school and not receiving schoolwork; not at school and receiving schoolwork.

At school	Not at school; not receiving schoolwork	Not at school; receiving schoolwork	Total
No FSM			
103	361	3308	3772
2.7%	9.6%	87.7%	100%
Yes FSM			
48	54	621	723
6.6%	7.5%	85.9%	100%
Total			
151	415	3929	4495
3.4%	9.2%	87.4%	100%

**Table 2.** Parental ethnic background by still attending school; not attending school and not receiving schoolwork; not at school and receiving schoolwork.

	At school	Not at school; not receiving schoolwork	Not at school; receiving schoolwork	Total
White	112	345	3244	3701
%	3.0	9.3	87.7	100
Indian	4	16	164	184
%	2.2	8.7	89.1	100
Pakistani/Bangladeshi	19	33	221	273
%	7.0	12.1	81.0	100
Black	10	6	131	147
%	6.8	4.1	89.1	100
Other	6	15	156	177
%	3.4	8.5	88.1	100
Total	151	415	3916	4482
%	3.4	9.3	87.4	100

Table 1 shows that 86% of those who received FSMs and 88% of those who did not receive FSMs were in our analysis, suggesting our sample was not selective in terms of whether students received FSMs or not. That said, among those not included in our analysis, those receiving FSMs were more likely to be at school. Therefore, the learning loss differences across socio-economic background for students excluded from analysis were not likely to be larger than for those included in analysis.

Furthermore, 3.4% of all children were still at school (Tables 1 and 2). The share with an Indian background was the lowest (2.2%); shares with a Pakistani and Bangladeshi background (7%) and a Black-Caribbean and Black-African background (6.8%) were the highest. About 9% did not attend school and did not receive any schoolwork from their schools, with the highest share representing children with Pakistani and Bangladeshi backgrounds (12%) and the lowest representing children with Black-Caribbean and Black-African backgrounds (4%). Pakistani and Bangladeshi students represented the lowest share (81%) who stayed at home during the school closure and received schoolwork (87.4% of the total). There were minor differences between White, Black-Caribbean and Black-African, Indian and Other ethnic groups, while those with Pakistani and Bangladeshi backgrounds were more likely to be in school and receiving no schoolwork if not in school.

### Variables

Our dependent variable was time children spent doing schoolwork provided by their school. The question was formulated as: ‘Thinking about the situation now, on an average day when they are doing schoolwork at home, how much time does [child name] spend on this?’ Answer categories were: (1) less than an hour; (2) 1 to 2 hours; (3) 2 to 3 hours; (4) 3 to 4 hours; (5) 4 to 5 hours; (6) 5 or more hours. We treated these categories as the value of their upper limit; this should be kept in mind when interpreting results. Table 3 shows the frequency distribution of all variables. Our dependent variable had an approximately normal distribution, with 24% of children spending 1–2 hours a day on home learning and another 24% spending 2–3 hours. Children in primary, secondary and higher secondary school spent, on average, 3.2 hours a day on schoolwork received from school, considerably lower than previous research has suggested (see Andrew et al., 2020).

**Table 3.** Descriptive statistics.

	Freq	%		Freq	%
*Time spent on home learning*			*School offline lessons*		
Less than an hour	347	11	None	233	7.4
1–2 hours	761	24.2	Less than 1 a day	327	10.4
2–3 hours	750	23.8	About 1 a day	571	18.1
3–4 hours	656	20.8	About 2 a day	614	19.5
4–5 hours	386	12.3	About 3 a day	684	21.7
5 or more hours	250	7.9	About 4 or more a day	721	22.9
*Sex*			*School online lessons*		
Male	1487	47.2	None	1741	55.3
Female	1476	46.9	Less than 1 a day	392	12.4
Unknown	187	5.9	About 1 a day	323	10.3
			About 2 a day	261	8.3
*Education phase*			About 3 a day	189	6
Primary: reception–KS2	1543	49	About 4 or more a day	244	7.8
Secondary: KS3–KS4	1265	40.2			
Higher-Secondary: KS5	342	10.9	*Work being checked by teacher*		
			No work provided	450	14.3
*Parents’ working arrangements*			None	548	17.4
Has no work	760	16.7	Less than half	424	13.5
Works from home	1465	32.1	Half or more	1728	54.9
Never work from home	2334	51.2			
			*Coronavirus symptoms in household*		
*Single parent*			No	2459	78.1
Couple	2745	87.1	Yes	691	21.9
Single	405	12.9			
			*Household size*		
*Parent education*			1	36	1.1
Degree	1711	54.3	2	116	3.7
A/AS level	300	9.5	3	499	15.8
GCSE or lower	1139	36.2	4	1516	48.1
			5	638	20.3
*FSM*			6	269	8.5
No	2645	84	7	34	1.1
Yes	505	16	8	21	0.7
			9	4	0.1
*Ethnicity*			10	8	0.3
White	2579	81.9	11	9	0.3
Indian	141	4.5			
Pakistani/Bangladeshi	187	5.9			
Black	110	3.5			
Other	133	4.2			
			Total	3150	100

Our independent variables for child characteristics were sex of the child and stage of education (primary, secondary, higher secondary). Sex is unknown for 187 children because they cannot be identified in the annual baseline Understanding Society dataset. We added these to our analysis as a separate category.

For parental characteristics, we included children’s eligibility for FSMs and parental/guardian education as socio-economic indicators. We also included whether parent/guardian was a single parent and parental ethnic background, using the parent (or guardian) who reported on the child(ren). As it is a common measure in education research in the UK, we used the information on whether child(ren) received FSMs at any time in January and/or February 2020 and parental education as indicators of socio-economic status. We operationalised the education of parent or guardian as degree, A/AS level (or other level 3 qualifications) or GCSE or lower. We amalgamated parental ethnic backgrounds into five categories: White, Pakistani or Bangladeshi, Black, Indian and Other, with the latter including all mixed backgrounds, Chinese, any other Asian and any other background. We also included a variable on whether parents worked; this had three categories: has no work, works from home (sometimes, often, always), never works from home. We added other variables such as ‘part-time work’ and ‘key-worker status’ but eliminated them, as the coefficients were not significant and did not contribute to the model fit.

We included three variables on schools’ provision of distance learning opportunities. The first was offline provision of lessons, asking how many offline lessons (e.g. worksheets, assignments, videos) the school provided for the child: none, less than one a day, about one a day, about two a day, about three a day, about four or more a day. A considerable proportion of children (7.4%) did not receive offline schoolwork from their school, and about 23% received four or more offline lessons each day. The second variable was provision of online lessons, asking how many online lessons or meetings the school provided, with the same answer categories as above. The majority (55.3%) did not receive any online distance teaching from the school; the range for the rest varied from less than once a day (12.4%) to four or more times a day (7.8%).

Parents/guardians were asked whether the teacher checked the schoolwork if it was sent in or uploaded. Answer categories were: no work provided, none of it, less than half, half or more, all of it. About 17% of the children did not have their work checked; about 55% had half or more of their work checked.

Finally, we controlled for whether somebody in the household showed COVID-19 symptoms and for household size.^1^

### Methods of Analysis

We used Ordinary Least Square (OLS) regression and built three nested models to show how the coefficients changed across models. Also known as hierarchical modelling, this method is used to explain relationships by using other explanatory variables. In this analysis, Model 1 was the base model, which included only control variables (children’s sex, children’s school stage, COVID-19 symptoms in the household, household size, whether parent works from home). Model 2 added the parental characteristics, thus showing the inequalities based on parental background (single parenthood, education of parent/guardian, child’s eligibility for FSM, parental ethnicity). Finally, Model 3 added the school provision variables to show how these were related to time children spent on home learning and to what extent they may explain some of the disparities revealed in Model 2.

We also ran our models using Ordered Logistic Regression (online Appendix B), but this did not change our conclusions. All regression models included cluster-corrected standard errors at the parent level, as there could be more than one child present for one parent/family.

## Results

### Descriptive Results

**Figures 1–4.**
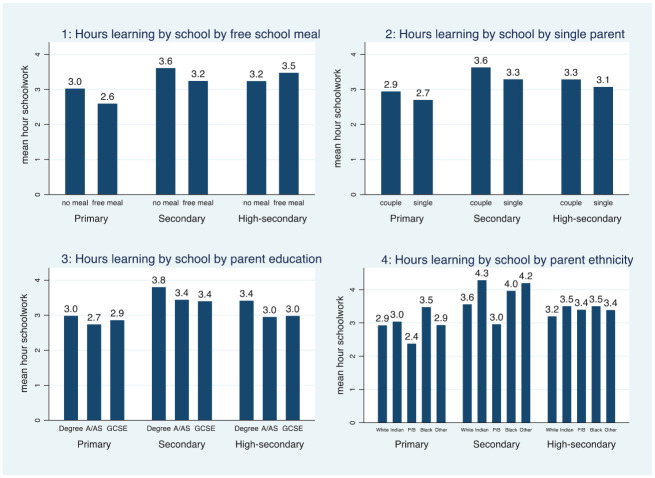
Hours of home learning by parental characteristics.

Our descriptive results showed some disparities in the time spent on schoolwork. Primary and secondary school children who received FSMs studied less but those at the higher secondary level studied more than their peers not receiving FSMs (Figure 1). Single-parent children spent fewer hours on schoolwork at home at all school stages (Figure 2). Secondary and higher secondary school children whose parents had a degree spent more time learning at home than those whose parents did not have a degree (Figure 3); primary school children with formally educated parents spent slightly more time on learning.

Considerable differences emerged for ethnic minority groups. Primary and secondary school children with Pakistani or Bangladeshi backgrounds spent substantially less time on home learning; those with a White majority background were the second lowest group (Figure 4). Primary school children with Black-Caribbean or Black-African backgrounds spent the most hours (3.5) on studying during the school closure. At the next stage, secondary school children with Indian and Other backgrounds spent the most time learning, 4.3 hours and 4.2 hours respectively. Overall, these results support Hypothesis 1 that the school closure would decrease learning, as 60% of students spent three hours or less on schoolwork.

### Regression Results

Multiple regression revealed significant differences between children’s individual, parental and school provision factors in the amount of time spent on schoolwork each day. Girls spent significantly more time on schoolwork than boys (Model 1, Table 4). Secondary school students spent significantly more time than higher secondary school students (Key stage 5 students); primary school children spent significantly less time. Children of parents who had no work or who never worked from home spent significantly less time on home learning than their peers whose parents mostly worked from home.

**Table 4.** Regression results on time spent on schoolwork.

	Model 1	Model 2	Model 3
	b/se	b/se	b/se
Sex of child (ref: male)			
female	0.345***	0.345***	0.245***
	0.05	0.05	0.045
unknown	0.127	0.183	0.092
	0.119	0.116	0.103
COVID-19 symptoms in household	0.083	0.04	0.064
	0.077	0.076	0.069
HH size	−0.027	−0.04	−0.032
	0.026	0.029	0.025
School phase (ref: higher secondary)			
primary	−0.259**	−0.238**	−0.319***
	0.091	0.092	0.082
secondary	0.423***	0.416***	0.042
	0.092	0.091	0.081
Parent works from home (ref: sometimes/always)			
Has no work at all	−0.377***	−0.174	−0.136
	0.097	0.1	0.093
Never works from home	−0.306***	−0.226***	−0.202***
	0.066	0.067	0.059
Single parent		−0.260*	−0.234**
		0.102	0.087
Parental education (ref: higher education degree or diploma)			
A/AS level		−0.226*	−0.198*
		0.099	0.079
GCSE or lower		−0.164*	−0.137*
		0.067	0.061
Free school meal eligibility		−0.266***	−0.153*
		0.079	0.069
Ethnicity (ref: White)			
Indian		0.349*	0.191
		0.151	0.131
Pakistani/Bangladeshi		−0.383**	−0.224
		0.138	0.127
Black-Caribbean/African/other		0.452**	0.312*
		0.168	0.138
Other ethnic background		0.124	0.063
		0.14	0.122
Offline schoolwork provision			0.297***
			0.018
Online schoolwork provision			0.182***
			0.016
Teacher checks schoolwork			0.193***
			0.023
_cons	3.333***	3.451***	1.597***
	0.143	0.154	0.161
*r*2	0.079	0.102	0.289
*N*	3150	3150	3150

Significance level: * < .05; ** < .01; *** < .001.

Model 1 explained about 8% of the variation in home learning; this increased to about 10% in Model 2 when the factors on parental background were added. Model 2 supported Hypothesis 2, except for some ethnic minority groups. That is, children whose parents had GCSE or lower-level qualifications spent significantly less time learning at home than those whose parents had a degree; pupils whose parents had A- or AS-level education spent even less time on home learning. Controlling for all other factors in Model 2 revealed children who received FSMs spent significantly less time studying at home than those who did not. Children from single-parent households spent significantly less time on home learning than their peers in couple households.

Model 2 compared time spent on home learning for children from different ethnic minority groups and those with a White background. Children with Indian and Black-Caribbean or Black-African ancestry spent significantly more time on schoolwork at home than their White peers, while students with a Pakistani or Bangladeshi background spent substantially less time, giving partial support for Hypothesis 2.

#### Schools’ Involvement in Home Learning

Provisions for distance teaching – the amount of offline learning material, online distance teaching, the checking of schoolwork – all significantly increased the time spent on schoolwork (Model 3, Table 4). In other words, the more often schools provided offline schoolwork, the more time children spent on learning at home. If schools taught online from a distance, student learning time significantly increased. It also increased when teachers checked schoolwork frequently.

Including schools’ involvement in home learning partly and for some parental background factors fully explained the differences in the impact of parental background on the amount of time students spent on schoolwork at home. When we compared the differences in regression coefficients of parental factors in Model 2 and Model 3, we found the negative impact of single parenthood on students’ home learning remained significant but taking schools’ distance learning provision into account considerably reduced its negative impact. Furthermore, indicators of schools’ distance teaching partly explained the effect found for lower parental education. The negative association for children’s FSM eligibility dropped by half when we controlled for distance teaching provision.

Except for children with Black-Caribbean or Black-African backgrounds, schools’ provision explained differences between White majority children and ethnic minority children. Children with an Indian background had better distance teaching provisions and spent more time on home learning (Model 3). Their schools provided offline and online teaching possibilities, and teachers checked schoolwork regularly. Therefore, when schools’ involvement was considered, their positive learning gap disappeared. The learning difference for children with Pakistani or Bangladeshi parents was also explained by schools’ learning provision (Model 3). That is, the learning gap of these children was likely produced by the schools they attended; more specifically, their schools less frequently offered distance learning materials and supervision.^2^ Model 3 greatly increased the explanatory power of the variance in students’ home learning time, rising from 10 to 29%, demonstrating that schools’ involvement and teaching provision better explained children’s home learning differences than parental background.

**Figure 5.**
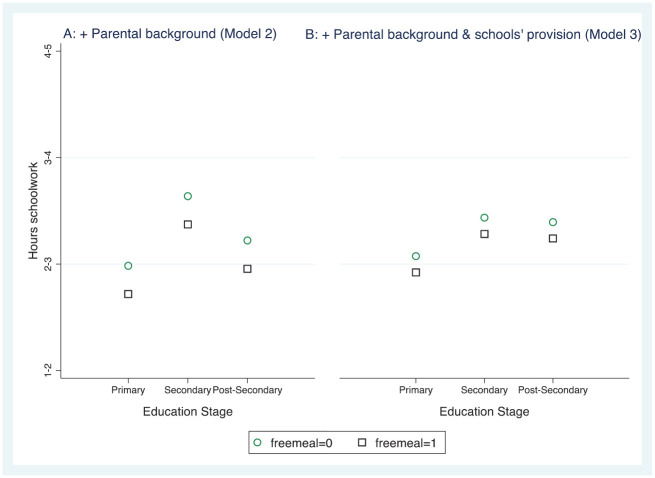
Marginal mean hours spent on home learning each day for children (not) receiving free school meals. *Note*: figures in A and B are from Models 2 and 3 respectively in Table 4.

Figure 5 depicts the study time of the children in primary, secondary and higher secondary education who received FSMs in early 2020 and those who did not. Part A shows the study hours, on average, for the different groups without controlling for school provision (Model 2, Table 4). Part B shows the distances between groups after taking school provision into account (Model 3). As the figure indicates, the home learning gap between students who did and did not receive FSMs decreased considerably when schools’ distance teaching provision was considered, yet some learning gap remained.

**Figure 6.**
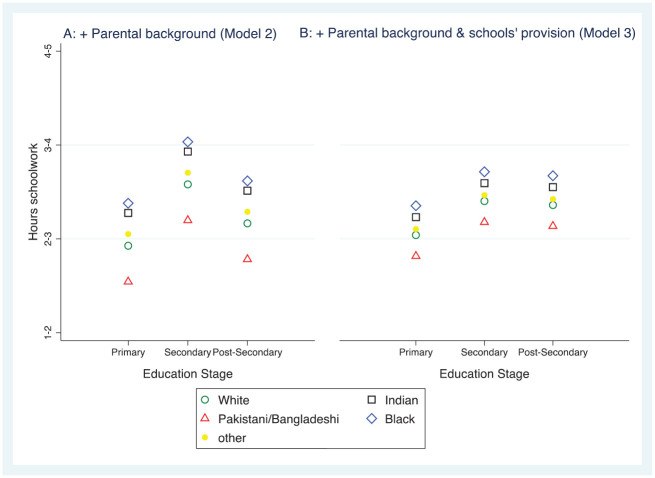
Marginal mean hours spent on home learning each day for children from ethnic minority groups. *Note*: figures in A and B are from Models 2 and 3 respectively, in Table 4.

Figure 6 shows differences in the study time of children in Models 2 and 3, this time with different ethnic backgrounds. Schools’ involvement in home learning substantially reduced differences based on ethnicity, supporting Hypothesis 3 that schools’ remote teaching provision would explain some educational inequalities.

## Discussion

Using the first wave of the Understanding Society COVID-19 data, we found children receiving schoolwork from their school spent, on average, 3.2 hours a day on home learning, supporting Hypothesis 1. Our findings also confirmed Hypothesis 2: children receiving FSMs, children whose parents had a lower level of formal education, especially those with A or AS level of education, and children in single-parent families spent less time on home learning than their comparators. Students with a Pakistani or Bangladeshi background spent the fewest hours on schoolwork. However, children with an Indian heritage spent significantly more time on schoolwork, in line with previous research on ethnic differences in educational attainment in the UK (Rothon, 2007).

Our analysis focused on those students who received at least some schoolwork. As there were proportionally more children with Pakistani or Bangladeshi backgrounds who were not in school and did not receive any schoolwork, the extent of the learning gap for this group might be even larger than our estimations. Children with Black-Caribbean or Black-African heritage spent the most hours on schoolwork, on average, across all ethnic groups and education stages, potentially reflecting the high aspirations of ethnic minority families (Fernández-Reino, 2016).

We found the learning gap attributed to family background was considerably reduced once the schools’ remote teaching was considered (confirming Hypothesis 3). During the pandemic, distance teaching provision was patchy and selective. Only some schools offered learning materials with tasks for students to do at home, and only some monitored completion of schoolwork and learning. We found schools’ online and offline distance teaching and homework checking not only significantly increased children’s spending time on home learning, but also explained the gap between those with Pakistani or Bangladeshi backgrounds and their White peers. Schools’ remote teaching provision also reduced the home learning gap between children with Black-Caribbean or Black-African and those with White heritage. We found Pakistani and Bangladeshi children studied less because their schools were less involved in ongoing learning during the school interruption. That is, if given the same learning opportunities, these students may not have differed from their comparators.

The schools that created disadvantage during the pandemic may have had fewer resources or been in areas more affected by the pandemic. Local and national authorities should invest in these schools to close the substantial learning gap that developed during the school interruptions and also prepare them to provide remote teaching during school interruptions in the future. The government should work to eliminate the digital divide between privileged and underprovided groups and prevent learning losses in future school closures.

Several research pathways remain to be pursued. First, spending more time on schoolwork may facilitate learning, but it does not necessarily equate to learning, and this limits our understanding of the actual learning differences. Children who are not provided with resources important to do well in school will likely learn less, even if they spend more time on schoolwork. Future research should investigate how time spent on schoolwork translates into learning for different groups and how resources – a computer, a study space, online and offline learning materials and so on – interact with the time spent on schoolwork to produce actual leaning.

Second, we do not know what the long-term consequences of home learning during the COVID-19 pandemic will be. Future research should examine learning gaps in test results in the coming years, as well as other life outcomes such as well-being and labour market transitions. Schools remained open for children of key workers, but we have limited understanding of whether learning during this period was different from the pre-pandemic period; research should focus on the learning and school experiences of the students who were in school during the school closure.

Third, studying the impact of the school closure on other kinds of learning, including non-formal and informal education spaces, may shed light on inequalities in education from a broader perspective. Fourth, where possible, researchers should re-examine our research questions with data collected from schools on their learning provision during the school closures merged with the student test scores rather than parental reports to overcome this limitation of our study. Such a design would also minimise potential bias that might have been introduced by response rates and potentially non-random participation stemming from survey design, such as financial incentives to encourage participation.

Our research makes two important contributions to the literature and has strong implications for policy development. First, education was most disrupted for children with lower socio-economic backgrounds and children with Pakistani and Bangladeshi heritage. Second, the inequalities documented across students from different socio-economic and ethnic backgrounds can be reduced by equipping students, schools and teachers with digital skills and resources. Our results highlight that the provision of learning differed across schools during the lockdown, and some students were penalised by receiving less teaching provision. Universally good quality remote teaching should be available to all students to tackle (at least some part of) the inequalities occurring during school closures.

## Supplemental Material

sj-docx-1-soc-10.1177_00380385221122444 – Supplemental material for Inequalities in Home Learning and Schools’ Remote Teaching Provision during the COVID-19 School Closure in the UK Click here for additional data file. Supplemental material, sj-docx-1-soc-10.1177_00380385221122444 for Inequalities in Home Learning and Schools’ Remote Teaching Provision during the COVID-19 School Closure in the UK by Sait Bayrakdar and Ayse Guveli in Sociology

## Notes

1. We included other variables to control relations in model building (online Appendix A). We tested the significance of relationships of other pre-existing variables, such as ‘relation to child’ (mother, father, guardian) and ‘school type’ (state/private). The coefficient for the ‘relation to child’ variable was insignificant or did not improve our model.

2. We ran several other models for robustness checks (online Appendix A). We tested models using school key stage information instead of school phase, parental occupational status and school type (private/state). Key stage information did not change our results. In the parental occupational status model, the association of low parental education and home learning became insignificant, as both are indicators of socio-economic status and highly correlated. Similarly, in the model with the school type information, the association between FSM and home learning became insignificant, as school type is likely to be a proxy for socio-economic class.
