# Supplementary material for: Inequalities in Home Learning and Schools’ Remote Teaching Provision during the COVID-19 School Closure in the UK
Source: Sociology. 2022 Oct 28;57(4):767–88. doi: 10.1177/00380385221122444 (PMC9618914; doi:10.1177/00380385221122444)
Supplement: sj-docx-1-soc-10.1177_00380385221122444 – Supplemental material for Inequalities in Home Learning and Schools’ Remote Teaching Provision during the COVID-19 School Closure in the UK [file sj-docx-1-soc-10.1177_00380385221122444.docx]

**Online Appendix**

Appendix A: Models for robustness checks

|  | Main model | Main model with key stages | Main model without parental education | Main model with occupational status | Main model with school type |
| --- | --- | --- | --- | --- | --- |
|  | b/se | b/se | b/se | b/se | b/se |
| Sex of child (ref: male) |  |  |  |  |  |
| *female* | 0.245*** | 0.245*** | 0.255*** | 0.244*** | 0.248*** |
|  | 0.045 | 0.044 | 0.04 | 0.045 | 0.045 |
| unknown | 0.092 | 0.081 | 0.054 | 0.095 | 0.312** |
|  | 0.103 | 0.102 | 0.089 | 0.102 | 0.115 |
| COVID19 symptoms in household | 0.064 | 0.057 | 0.064 | 0.063 | 0.063 |
|  | 0.069 | 0.068 | 0.059 | 0.069 | 0.069 |
| HH size | -0.032 | -0.036 | -0.041 | -0.029 | -0.033 |
|  | 0.025 | 0.025 | 0.022 | 0.025 | 0.025 |
| School phase (ref: higher secondary) | |  |  |  |  |
| *primary* | -0.319*** |  | -0.325*** | -0.314*** | -0.320*** |
|  | 0.082 |  | 0.074 | 0.083 | 0.082 |
| *secondary* | 0.042 |  | 0.015 | 0.055 | 0.005 |
|  | 0.081 |  | 0.073 | 0.081 | 0.082 |
| School phase (ref: KS2) |  |  |  |  |  |
| *Reception year (age 4)* |  | -0.634*** |  |  |  |
|  |  | 0.094 |  |  |  |
| *KS1: years 1-2 (age 5-7)* |  | -0.370*** |  |  |  |
|  |  | 0.069 |  |  |  |
|  |  | 0.304*** |  |  |  |
| *KS3: years 7-9 (age 11-14)* |  | 0.059 |  |  |  |
|  |  | 0.105 |  |  |  |
| *KS4: years 10-11 (age 14-16)* |  | 0.076 |  |  |  |
|  |  | 0.196* |  |  |  |
| *KS5: years 12-13 (age 16-18)* |  | 0.085 |  |  |  |
| Parent working from home (ref: sometimes/always) |  |  |  |  |  |
| *Not working at all* | -0.136 | -0.157 | -0.135 | -0.114 | -0.149 |
|  | 0.093 | *0.092* | 0.081 | 0.106 | 0.093 |
| *Never from home* | -0.202*** | -0.201*** | -0.216*** | -0.171** | -0.202*** |
|  | 0.059 | 0.059 | 0.053 | 0.062 | 0.059 |
| Single parent | -0.234** | -0.278** | -0.251** | -0.225** | -0.250** |
|  | 0.087 | 0.087 | 0.08 | 0.087 | 0.087 |
| Parental education (ref: higher education degree or diploma) |  |  |  |  |  |
| *A/AS level* | -0.198* | -0.197* |  | -0.192* | -0.195* |
|  | 0.079 | 0.078 |  | 0.081 | 0.079 |
| *GCSE or lower* | -0.137* | -0.158** |  | -0.102 | -0.140* |
|  | 0.061 | 0.06 |  | 0.064 | 0.061 |
| Free school meal eligibility | -0.153* | 0.033 | -0.202** | -0.150* | -0.081 |
|  | 0.069 | 0.076 | 0.067 | 0.069 | 0.072 |
| Ethnicity (ref: White) |  |  |  |  |  |
| *Indian* | 0.191 | 0.211 | 0.221 | 0.193 | 0.188 |
|  | 0.131 | 0.131 | 0.121 | 0.132 | 0.131 |
| *Pakistani/Bangladeshi* | -0.224 | -0.22 | -0.215 | -0.214 | -0.213 |
|  | 0.127 | 0.124 | 0.116 | 0.125 | 0.126 |
| *Black-Caribbean/African/other* | 0.312* | 0.310* | 0.346** | 0.321* | 0.306* |
|  | 0.138 | 0.136 | 0.132 | 0.135 | 0.138 |
| *Other ethnic background* | 0.063 | 0.051 | 0.164 | 0.078 | 0.039 |
|  | 0.122 | 0.12 | 0.122 | 0.123 | 0.121 |
| Offline schoolwork provision | 0.297*** | 0.290*** | 0.297*** | 0.296*** | 0.291*** |
|  | 0.018 | 0.018 | 0.017 | 0.018 | 0.018 |
| Online schoolwork provision | 0.182*** | 0.180*** | 0.180*** | 0.184*** | 0.176*** |
|  | 0.016 | 0.016 | 0.015 | 0.016 | 0.016 |
| Teacher checks schoolwork | 0.193*** | 0.184*** | 0.182*** | 0.191*** | 0.190*** |
|  | 0.023 | 0.023 | 0.021 | 0.023 | 0.023 |
| Parental occupational status (Ref: management & professional) |  |  |  |  |  |
| *intermediate* |  |  |  | 0.017 |  |
|  |  |  |  | 0.085 |  |
| *small employers & own account* |  |  |  | -0.196 |  |
|  |  |  |  | 0.116 |  |
| *lower supervisory & technical* |  |  |  | -0.019 |  |
|  |  |  |  | 0.143 |  |
| *semi-routine & routine* |  |  |  | -0.228** |  |
|  |  |  |  | 0.078 |  |
| *missing* |  |  |  | -0.062 |  |
|  |  |  |  | 0.093 |  |
| School type (ref: state) |  |  |  |  |  |
| *private* |  |  |  |  | 0.202 |
|  |  |  |  |  | 0.158 |
| *missing* |  |  |  |  | -0.254*** |
|  |  |  |  |  | 0.065 |
| _cons | 1.597*** | 1.477*** | 1.612*** | 1.604*** | 1.688*** |
|  | 0.161 | 0.152 | *0.144* | 0.162 | 0.163 |
| r2 | 0.289 | 0.301 | 0.279 | 0.292 | 0.293 |
| N | 3150 | 3150 | 3867 | 3150 | 3150 |

| Appendix B: Results from ordered logistic regression | | | |
| --- | --- | --- | --- |
|  | Model 1 | Model 2 | Model 3 |
|  | b/se | b/se | b/se |
| Sex of child (ref: male) | |  |  |
| *female* | 0.465*** | 0.468*** | 0.381*** |
|  | 0.066 | 0.066 | 0.068 |
| unknown | 0.154 | 0.249 | 0.073 |
|  | 0.153 | 0.153 | 0.155 |
| COVID19 symptoms in household | 0.104 | 0.035 | 0.082 |
|  | 0.101 | 0.101 | 0.104 |
| HH size | -0.047 | -0.057 | -0.041 |
|  | 0.034 | 0.039 | 0.038 |
| School phase (ref: higher secondary) | | |  |
| *primary* | -0.251* | -0.250* | -0.432*** |
|  | 0.122 | 0.125 | 0.122 |
| *lower secondary* | 0.596*** | 0.578*** | 0.056 |
|  | 0.124 | 0.125 | 0.12 |
| Parent working from home (ref: sometimes/always) | | |  |
| *Not working at all* | | -0.260* | -0.197 |
|  |  | 0.132 | 0.138 |
| *Never from home* | | -0.316*** | -0.304*** |
|  |  | 0.089 | 0.09 |
| Single parent |  | -0.381** | -0.350** |
|  |  | 0.14 | 0.131 |
| Parental education (ref: higher education degree or diploma) | | | |
| *A/AS level* |  | -0.275* | -0.281* |
|  |  | 0.131 | 0.119 |
| *GCSE or lower* |  | -0.197* | -0.179* |
|  |  | 0.087 | 0.09 |
| Free school meal eligibility | | -0.381*** | -0.229* |
|  |  | 0.106 | 0.106 |
| Ethnicity (ref: white) | |  |  |
| *Indian* |  | 0.460* | 0.271 |
|  |  | 0.199 | 0.203 |
| *Pakistani/Bangladeshi* | | -0.519** | -0.36 |
|  |  | 0.186 | 0.19 |
| *Black-Caribbean/African/other* | | 0.599** | 0.506* |
|  |  | 0.219 | 0.202 |
| *Other ethnic background* | | 0.158 | 0.117 |
|  |  | 0.182 | 0.179 |
| Offline schoolwork provision | |  | 0.476*** |
|  |  |  | 0.031 |
| Online schoolwork provision | |  | 0.275*** |
|  |  |  | 0.024 |
| Teacher checks schoolwork | |  | 0.304*** |
|  |  |  | 0.036 |
|  |  |  |  |
| cut1 | -2.009*** | -2.517*** | 0.108 |
|  | 0.189 | 0.22 | 0.252 |
| cut2 | -0.493** | -0.967*** | 1.881*** |
|  | 0.183 | 0.212 | 0.253 |
| cut3 | 0.528** | 0.087 | 3.128*** |
|  | 0.183 | 0.21 | 0.26 |
| cut4 | 1.591*** | 1.178*** | 4.399*** |
|  | 0.186 | 0.211 | 0.267 |
| cut5 | 2.705*** | 2.311*** | 5.664*** |
|  | 0.196 | 0.219 | 0.28 |
|  |  |  |  |
| N | 3150 | 3150 | 3150 |
